# Mechanical Behavior of Adhesively Bonded Joints Under Tensile Loading: A Synthetic Review of Configurations, Modeling, and Design Considerations

**DOI:** 10.3390/ma18153557

**Published:** 2025-07-29

**Authors:** Leila Monajati, Aurelian Vadean, Rachid Boukhili

**Affiliations:** 1 Mechanical Engineering Department, Polytechnique Montréal, Montreal, QC H3T 1J4, Canada; leila.monajati@polymtl.ca (L.M.); aurelian.vadean@polymtl.ca (A.V.); 2 Research Centre for High Performance Polymer and Composite Systems (CREPEC), Montreal, QC, Canada

**Keywords:** adhesively bonded joints, tensile performance, joint configurations, finite element analysis (FEA), cohesive zone modeling (CZM), failure mechanisms, nano-enhanced adhesives, structural optimization, damage tolerance

## Abstract

This review presents a comprehensive synthesis of recent advances in the tensile performance of adhesively bonded joints, focusing on applied aspects and modeling developments rather than providing a full theoretical analysis. Although many studies have addressed individual joint types or modeling techniques, an integrated review that compares joint configurations, modeling strategies, and performance optimization methods under tensile loading remains lacking. This work addresses that gap by examining the mechanical behavior of key joint types, namely, single-lap, single-strap, and double-strap joints, and highlighting their differences in stress distribution, failure mechanisms, and structural efficiency. Modeling and simulation approaches, including cohesive zone modeling, extended finite element methods, and virtual crack closure techniques, are assessed for their predictive accuracy and applicability to various joint geometries. This review also covers material and geometric enhancements, such as adherend tapering, fillets, notching, bi-adhesives, functionally graded bondlines, and nano-enhanced adhesives. These strategies are evaluated in terms of their ability to reduce stress concentrations and improve damage tolerance. Failure modes, adhesive and adherend defects, and delamination risks are also discussed. Finally, comparative insights into different joint configurations illustrate how geometry and adhesive selection influence strength, energy absorption, and weight efficiency. This review provides design-oriented guidance for optimizing bonded joints in aerospace, automotive, and structural engineering applications.

## 1. Introduction

Adhesive bonding is a critical joining technique in modern engineering, offering advantages in weight reduction and more uniform stress distribution compared to mechanical fastening [1,2]. However, predicting and improving the tensile performance of bonded joints remains challenging due to complex stress distributions and diverse failure modes. Campilho [3] provides an overview of design challenges and limitations for adhesive joints, highlighting the need for improved design methodologies. Moreover, various joint configurations, including single/double lap, scarf, strap, and butt joints, can be used to connect composite structures, each offering unique advantages depending on load distribution, space constraints, and material properties [4]. Recent research has, therefore, focused on understanding how various factors, including joint configuration, material properties, geometric modifications, defects, and modeling and manufacturing techniques, influence the strength and failure behavior of adhesively bonded joints [5]. 

Considering the diverse structural and service requirements across engineering sectors, a wide range of adhesively bonded joint configurations has been developed to address different mechanical, manufacturing, and environmental constraints. Each configuration exhibits unique load transfer characteristics, stress distributions, and failure modes. However, despite the large body of experimental and numerical research on individual joint types or modeling techniques, there is still a lack of integrated resources that systematically compare joint configurations, consolidate advances in simulation methods, and evaluate material and geometric design strategies under tensile loading conditions. Such a synthesis is essential to providing engineers with clear guidance that unifies mechanical performance characteristics, modeling approaches, and design considerations. 

A central question motivating this review is, “**What defines an optimal bonded joint under tensile loading?**” Answering this question requires a nuanced understanding of how geometry, material behavior, and stress conditions interact. Specifically, an optimal joint should

- Minimize local stiffness gradients to reduce stress concentrations;
- Promote shear-dominated rather than peel-dominated loading;
- Exhibit predictable and progressive damage evolution; and
- Avoid brittle failure by incorporating ductility or enhanced damage tolerance.

**Figure 1:**
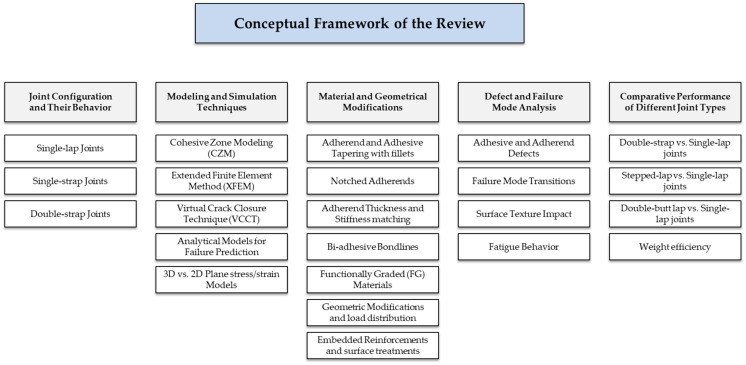
Conceptual framework guiding the thematic synthesis of the literature review.

The aim of this review is to provide a comprehensive and comparative synthesis of the mechanical performance of adhesively bonded joints under tensile loading. Specifically, this work seeks to (i) critically analyze the behavior of common joint configurations, such as single-lap, single-strap, and double-strap joints; (ii) review recent advances in modeling and simulation approaches, including cohesive zone modeling (CZM), extended finite element method (XFEM), and virtual crack closure technique (VCCT), to predict failure mechanisms and load capacity; (iii) evaluate the influence of material and geometric modifications (e.g., bi-adhesive bonds, adherend tapering, functionally graded materials) on improving joint strength and damage tolerance; (iv) defect and failure mode analysis; and (v) comparative Performance of Different Joint Types. The conceptual framework guiding the thematic organization of this review is presented in Figure 1. The ultimate purpose is to offer design-oriented guidance that supports engineers and researchers in selecting, optimizing, and developing adhesively bonded joints for high-performance applications in aerospace, automotive, and structural engineering. The intended audience is engineers and researchers seeking a comprehensive understanding of recent advancements in the tensile analysis of adhesive joints.

## 2. Joint Configurations and Their Behavior

**Figure 2:**
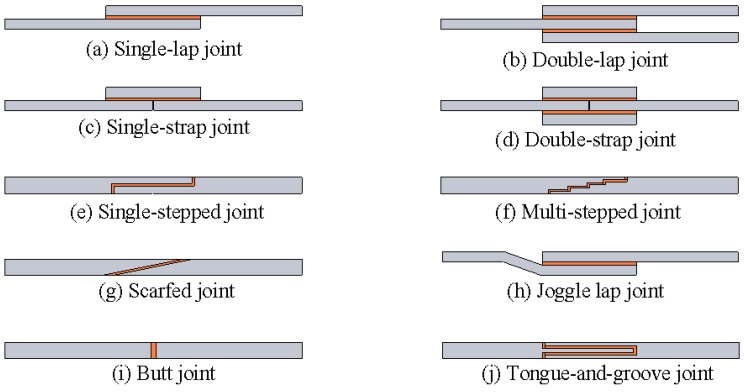
Schematic representation of various bonded joint configurations (The orange region represents the adhesive layer).

Adhesively bonded joints come in various configurations, each with distinct load distribution characteristics. The most common types, including single/double-lap and single/double-strap joints, and more specialized variants, such as single/multi-stepped, scarfed, joggle, butt, and tongue-and-groove joints, are shown in Figure 2. No single configuration is universally best; each has advantages, depending on the use case and materials. This section reviews key findings for three widely studied configurations under tensile loading: single-lap, single-strap, and double-strap joints.

### 2.1. Single-Lap Joints (SLJ)

Single-lap joints (SLJs) (shown in Figure 2a) are one of the simplest and most widely studied configurations. A SLJ consists of two adherends overlapping in a single plane with adhesive in between, creating an eccentric load path that induces both shear and peel stresses. Under tensile load, the offset between adherends causes secondary bending and the joint tends to open at the ends, concentrating peel stress at the ends of the bonded area. As a result, failure in SLJs often initiates at one of the overlap ends, where the combination of shear and peel stress is highest. This eccentricity is a key weakness of SLJs, making them sensitive to adherend bending stiffness and adhesive toughness.

#### 2.1.1. Experimental Observations

**Figure 3:**
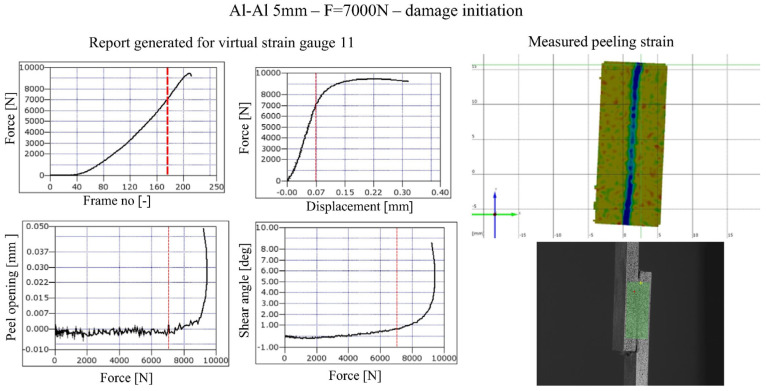
An example of a generated DIC report at the onset of damage in an SLJ configuration [9]. The four presented plots illustrate the corresponding frame number for a given applied force, the variation in locally measured vertical displacement with increasing load, peeling strain distribution, and shear angle evolution as a function of the applied load.

Numerous studies have characterized SLJ behavior experimentally. Digital Image Correlation (DIC) techniques have been widely used to capture full-field strain in SLJs. An example of data obtained from DIC measurements is shown in Figure 3. Comer et al. [6] applied DIC to composite SLJs and confirmed highly non-uniform strain distributions. Kavdir and Aydin [7] compared strain distributions obtained from both experimental and numerical analyses. They concluded that three-dimensional (3D) DIC provides more accurate results than its 2D counterpart, emphasizing careful specimen preparation to obtain reliable adhesive properties. It was also noted that increasing the adhesive layer thickness led to more reliable results, likely because the larger bonded area was better captured and defined by the software. DIC-based studies have also shown that real SLJs often fail at lower loads than ideal simulations predict because actual adhesives contain micro-defects and cure shrinkage stresses. Kumar et al. [8] reported that micro-voids and imperfections act as stress raisers, explaining why finite element models of “perfect” SLJs tend to overestimate strength.

#### 2.1.2. Key Factors and Findings

Several geometric and material parameters strongly affect SLJ performance. Overlap length is one primary factor: a longer overlap spreads the load, reducing peak stresses. However, beyond a certain effective bond length, additional overlap yields diminishing returns [10]. Ye et al. [11] observed a transition in failure mode with increasing overlap. Short overlaps failed in the adhesive, whereas sufficiently long overlaps caused delamination in composite adherends. Adherend stiffness and thickness are also important. Thicker or stiffer adherends reduce bending deformation, which can raise strength up to a point. Gültekin et al. [12] demonstrated that both the moment and flexural rigidity of the adherends play a significant role in determining the strength of single-lap joints. Higher flexural rigidity was found to reduce peel-induced deformation, thereby increasing the joint’s overall load capacity. Aydin et al. [13] showed that increasing the adherend thickness in aluminum single-lap joints (SLJs) promotes the transfer of shear stress and strain from the overlap edges toward the center, primarily due to a reduction in peel effects. This more balanced stress distribution contributes significantly to the increased joint strength observed at greater adherend thicknesses, given a constant overlap length.

If the two adherends have dissimilar stiffness, the more flexible side will dominate deformation and typically govern failure. For the strongest joint, adherends should be as stiff as possible and similar to each other in stiffness. This insight is echoed by Ozel et al. [14], who conducted a comparative study on SLJs with different adherend materials, providing insight into how adherend properties affect joint strength. They considered various material pairings, including metals and composites, and observed that joints with stiffer adherends generally exhibited higher shear strength and more uniform stress distributions due to carrying a greater portion of the load. However, in joints with highly mismatched adherends (e.g., one very stiff and one very flexible adherend), the overall joint strength was governed by the less-stiff adherend, which would deform more and concentrate stress. In other words, the joint is only as strong as its more compliant adherend [15]. 

#### 2.1.3. Mechanical Behavior and Failure Modeling

Failure modes in SLJs can be cohesive (through the adhesive), interfacial (adhesive-substrate debonding), or in the adherend (e.g., delamination in composites or yielding in ductile metals). Often, a combination occurs. Stuparu et al. [9] examined both similar and dissimilar material SLJs. Dissimilar joints composed of aluminum and carbon fiber adherends showed lower strength, primarily due to delamination in the composite adherend induced by peel stresses. Standard simulations, which overlooked internal composite damage, tended to over-predict strength due to an incomplete representation of delamination. To enhance accuracy, they recommended using refined laminate modeling techniques. In a related study, Taib et al. [16] performed finite element analysis of single and joggled lap joints with composite adherends, revealing how geometric discontinuities like joggles can redistribute stress and mitigate critical peel zones, thereby improving joint durability. Kupski et al. [17] demonstrated that the ply layup sequence in a composite adherend strongly influences the failure mechanism: with a 0° fiber layer at the bond interface, failure was more likely within the adhesive (cohesive failure) because the adherend could carry load well, whereas a 90° surface ply (fibers perpendicular to load) made the interface weaker and led to composite adherend failure. Their study further concluded that increasing the bending stiffness of the adherend enhances the joint’s tensile strength by delaying damage initiation. However, achieving higher ultimate load capacities requires a stacking sequence that promotes crack propagation within the composite adherend. As another example, Luo et al. [18] observed that SLJs with thick, stiff adherends tended to fail cohesively in the adhesive, whereas joints with thinner, more flexible adherends often failed due to a mix of laminate peeling in the composite and fiber tearing. This occurs because flexible adherends bend more, increasing peel stress that can strip fibers from the composite surface. In the case of defective adhesives, a mixed failure mode was found to be dominant. Tang et al. [19] studied SLJs focusing on how bondline thickness affects failure modes. They observed that failure in thick adherend joints typically initiated at the adherend adhesive interface, followed by interlaminar (delamination) failure in the composite adherend’s first ply near the interface. Interestingly, their experiments showed that reducing the bondline thickness increased joint strength. Thinner adhesive layers promoted stronger joints in their case, likely because the thicker adhesives introduced more compliance and greater peel stresses under load.

Given these considerations, many research efforts on SLJs aim to reduce peel stress and achieve a more uniform stress distribution. Common geometric modifications have proven effective and are discussed in Section 4.1. In summary, the single-lap joint is a convenient and widely used configuration, but its inherent eccentricity makes it prone to peel-driven failure. Research indicates that through careful design and adhesive selections, the performance of SLJs can be substantially improved.

### 2.2. Single-Strap Joints

Single-strap joints (shown in Figure 2c) involve two co-linear adherends joined end-to-end with a third piece (the strap) bonded on one side across the joint. Compared to single-lap joints, single-strap joints are symmetric through the thickness, which eliminates out-of-plane bending caused by eccentric loading. However, because the reinforcement strap is bonded on only one side, the assembly is asymmetric about the adhesive layer, causing unbalanced load transfer between the strap-reinforced side and the unreinforced side and thereby inducing a bending moment across the bonded interface. In essence, a relatively long single-strap joint behaves somewhat like two single-lap joints in series, while shortening the specimen increases the sensitivity of the results to boundary conditions [20].

#### 2.2.1. Stress Distribution and Design Parameters

**Figure 4:**
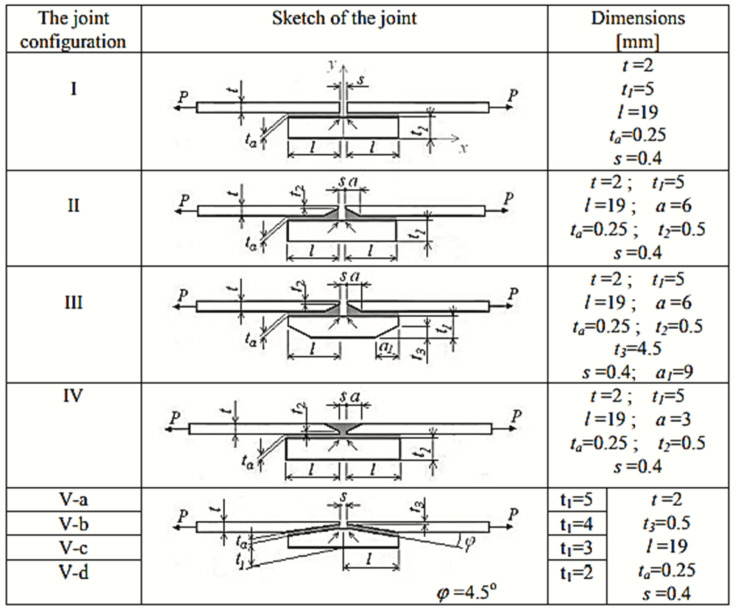
Configurations considered for single-strapped bonded joints [21] with arrows indicating regions of highest stress. Configurations II and III were found to provide the greatest joint strength.

Sandu et al. [21] analyzed the tapering strap and adherend ends and found the highest stress concentrations in the adhesive, indicated by arrows shown in Figure 4. Lower tapered adherend ends (designs II and III) were shown to reduce stress concentration significantly, achieving the highest joint strengths. Therefore, carefully shaping the strap/adherend interface in that region is key to performance. Jiang et al. [22] performed finite element analysis on a carbon/epoxy composite single-strap joint and examined how joint dimensions and composite layup affected strength. They found that using a 0° fiber orientation in the layers of the adherends and strap adjacent to the adhesive maximized the joint strength. They also studied the optimal adhesive thickness: a thinner bondline increased stress concentration, whereas an overly thick bondline caused relative separation of the adhesive elements under shear force, leading to failure when the deformation exceeded the adhesive’s capacity. An excessively thick bondline did not further increase strength and could cause difficulty in controlling surface quality and internal defects. Similarly, increasing the strap overlap length improved strength by distributing load, but this approach, while improving strength, is unsuitable for lightweight design and can contribute to composite failure. This suggests that for weight-sensitive design (e.g., aerospace), an efficient length exists that balances strength along weight and material behavior.

#### 2.2.2. Strap Geometry Effects

Sandu et al. [20] experimentally compared single-strap joints with either square or tapered adherends within the overlap region. The tapered configuration exhibited significantly lower stress concentrations at the adhesive ends and higher joint strength. Tapering effectively spreads the load over a slightly longer region and avoids a sharp adhesive end. They also observed that when the strap overlap was relatively long, the joint behaved like two independent single lap joints in series. But as the overlap was shortened, the joint’s behavior became more sensitive to boundary conditions. This suggests that for testing purposes, an optimized joint length exists where the specimen is long enough to be representative yet short enough to be practical. Li et al. [23] addressed this by deriving an optimized specimen size for single-strap joints. By maintaining geometric similarity and appropriate non-dimensional parameters, they designed a lab-scale test that efficiently emulates the behavior of larger structures, which is useful for experimental efficiency. Their approach can be extended to other butt joint configurations with varying material properties.

Overall, single-strap joints benefit from design practices similar to SLJs: use of tapered or filleted geometry to reduce edge stresses, use of ductile adhesives to absorb peel forces, and fiber alignment with the load to strengthen the interface. Additionally, it is crucial to consider the effect of load eccentricity introduced by the single-sided strap, which induces a bending moment in the joint assembly. One strategy to mitigate that bending is the double-strap joint, discussed next, which bonds straps on both sides, ensuring more balanced load distribution.

### 2.3. Double-Strap Joints

Double-strap joints (shown in Figure 2b) consist of two cover plates (straps), one on each side of a butt joint, creating a symmetric configuration. This structural symmetry eliminates bending: the load is distributed evenly through the two adhesive layers, and the load path aligns with the joint’s mid-plane, preventing the generation of eccentric moment in an ideal, perfectly symmetric configuration. As a result, double-strap joints can carry higher loads than single-strap joints and are commonly used to repair or reinforce structural members.

#### 2.3.1. Load Transfer and Effective Bond Length

With two adhesive layers sharing the load, double-strap joints distribute stress more uniformly. An important design parameter is the effective bond length, the minimum overlap length beyond which increasing it does not enhance joint strength, resulting instead in added mass without functional benefit. Fawzia et al. [24] used this concept for steel plates bonded with CFRP patches, noting that beyond a certain length, the load transfer approached a plateau. Kadhim [25] later studied factors affecting effective bond length in steel–CFRP double-strap joints and found it to be relatively insensitive to many parameters: variations in CFRP patch and adhesive or steel thicknesses did not drastically change the required overlap length, whereas the number of CFRP layers was shown to have a notable effect on the effective bond length. For design, this means that one can estimate a development length for the adhesive beyond which the additional length is wasted.

#### 2.3.2. Geometric Effects

Çitil et al. [26] studied a double-strap configuration with an embedded patch. They found that making the embedded patch thinner improved load capacity because a thinner patch caused less strain mismatch between the patch and adhesive at the interfaces. Increasing the adherend thickness also raised strength, whereas extending the overlap length did not contribute to any significant improvement in joint strength. In another research, Çitil et al. [27] investigated the effect of inserting an intermediate component into the gap between patches in a double-strap joint. Tests showed that the intermediate piece was actually detrimental since joints without it had higher strength. The extra interfaces introduced by the spacer became weak points without offering benefits. Their optimal recommendation was to avoid using the intermediate part, maximize overlap length and adherend thickness, and minimize gap length and patch thickness within practical limits. Another geometric nuance is the strap to adherend stiffness balance. Paygozar et al. [28] varied the strap material and dimensions and found that the strap material had little effect on ultimate load. However, it had a significant impact on the elongation at failure, as the elongation increased when the strap material was changed from aluminum to glass-fiber reinforced polymer (GFRP). Regarding geometry, a longer patch (strap) increased failure load, but if the patch was too long, the adherends or the patch itself could start to fail before the adhesive. Conversely, reducing patch thickness made the patch more likely to yield or tear before cohesive failure could occur. Thus, there is an optimal patch thickness: too thick adds weight and stiffness without much strength benefit, too thin, and the patch might rupture first.

#### 2.3.3. Failure Modes and Comparative Performance

**Figure 5:**
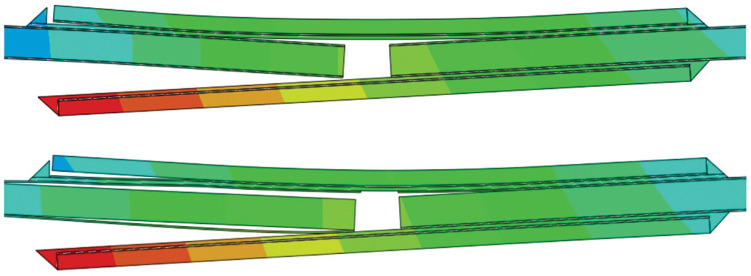
Finite element visualization of the displacement field and delamination propagation in the DSJ specimen [29]. The upper image depicts the simulated DSJ-crack model with only CZM applied to the adhesive layer, while the lower image includes both cohesive failure in the adhesive and delamination in the CFRP components. Delamination initiates near the pre-existing crack in the parent CFRP laminate and propagates into the strap laminate. Damage accumulation is concentrated on the pre-cracked side, highlighting the asymmetric failure behavior observed in the simulation.

Jokinen et al. [29] showed that if a crack or debond is present on one side only, the intact side continues to bear load, leading to an unbalanced but not immediate failure, as shown in Figure 5. Nevertheless, overall strength and stiffness are reduced, and ultimately, the flawed side’s failure will precipitate total failure once its load can no longer be redistributed. Tsai and Morton [30] observed that for composite straps and adherends, a unidirectional layup led to cohesive failure, whereas a quasi-isotropic layup led to delamination of the first ply at the strap ends. The difference was attributed to longitudinal stiffness: unidirectional carbon is very stiff along the fiber direction, resulting in a sharp load transfer into the adhesive, which then fails cohesively, while a quasi-isotropic laminate is less stiff at the interface and more prone to internal failure (ply delamination) under the complex stress state. Akpınar’s study [31,32] on composite patches bonded to aluminum confirmed that fiber orientation significantly influences joint performance, emphasizing the effectiveness of hybrid patching techniques. Using composite instead of aluminum patches greatly increased the joint’s load capacity since composites can be tailored for high strength in the load direction. However, not all composite patches are equal, with patches with a 90° orientation exhibiting significantly lower strength. Patches with ±45° layups caused an unusual peel-stress distribution. Instead of peaking at the ends, the peak peel stress shifted toward the center of the overlap. This was attributed to the anisotropic load coupling in angled plies. Regardless of orientation, peel stresses were identified as major contributors to failure in double-strap joints. Reducing peel stress is, thus, key to improving joint strength.

A striking finding by Azeem et al. [33] was that a mismatched double-strap joint (different strap placements on each side) could actually outperform a perfectly symmetric joint. In their tests on metal/composite laminate straps, the mismatched joints had higher strength, absorbed more fracture energy, and showed greater elongation at break than matched joints, even though both failed by similar mixed adhesive/cohesive modes. In other words, the asymmetry caused one side of the bond to take load longer and fail slightly later, delaying full fracture. This is somewhat counter-intuitive since symmetry is usually preferred for uniform load share, but it suggests that staged failure can be beneficial for toughness.

Dynamic loading is another consideration. Al-Zubaidy et al. [34] studied double-strap joints under rapid tensile loading and found some differences compared to quasi-static behavior. Notably, when using multiple layers of CFRP in the strap, their finite element model, which used an equivalent laminate with homogenized macroscopic properties, overpredicted strength and did not capture the delamination between layers that occurred in experiments. This points out that when modeling multi-layer composite straps, one must account for individual layers or use an accurate cohesive model for layers if delamination is a potential failure mode. It also highlights the general need for careful validation of models under dynamic loading conditions.

In summary, double-strap joints generally exhibit high strength and damage tolerance. Their design should ensure that both adhesives and straps carry loads as evenly as possible up to failure. Key design points include sufficient overlap to reach effective bond length, avoiding unnecessary gaps or inserts, and tailoring the patch layup for the load. With these considerations, double-strap joints can achieve excellent performance. 

Table 1 summarizes the joint configurations examined in this section, highlighting their key characteristics and design considerations.

## 3. Modeling and Simulation Techniques

**Figure 6:**
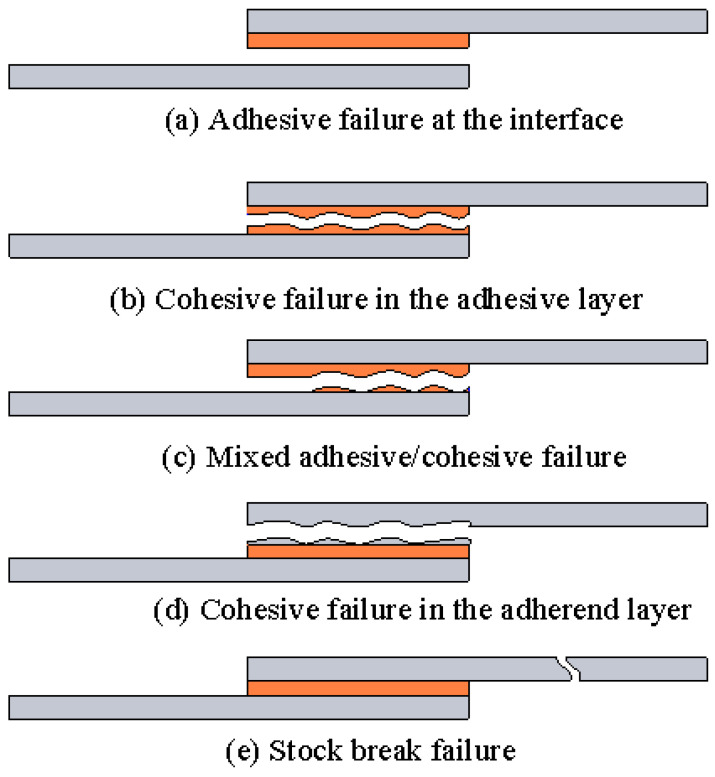
Common types of bonding failure, including (**a**) adhesive failure at the interface, (**b**) cohesive failure in the adhesive layer, (**c**) mixed adhesive/cohesive failure, (**d**) cohesive failure in the adherend layer, and (**e**) stock-break failure.

Predictive modeling of adhesive joints has evolved from simple analytic formulas to sophisticated numerical methods. Accurate models are crucial for designing joints and understanding failure processes, as physical testing of every scenario is costly. The challenge in modeling is capturing the nonlinear behavior of adhesives and the multiple possible failure modes, including adhesive failure at the interface, cohesive failure in the adhesive layer, mixed adhesive/cohesive failure, cohesive failure in the adherend layer, and stock break failure, as shown in Figure 6. This section reviews key simulation techniques and failure criteria, including cohesive zone modeling (CZM), extended finite element method (XFEM), virtual crack closure technique (VCCT), as well as analytical approaches. It also discusses the differences between 2D and 3D joint modeling. More detailed failure theories are presented in the review by Tserpes et al. [35].

### 3.1. Cohesive Zone Modeling (CZM)

**Figure 7:**
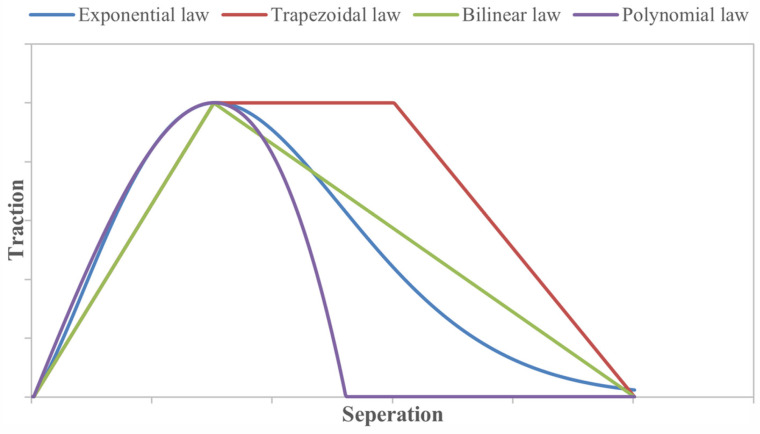
Common traction–separation laws used for CZM, including bilinear, exponential, trapezoidal, and polynomial laws.

CZM is one of the most widely used approaches for simulating progressive damage and failure in bonded joints. In a CZM, the adhesive layer or interface is governed by a traction–separation law, a relationship between the stress across the bond and the separation of the faces. Before failure, the elements carry load (traction) as a function of separation, usually linear elastic up to a peak stress, followed by softening to represent damage until complete separation. The area under the traction–separation curve is the fracture energy (toughness) of the adhesive. Several traction–separation laws are commonly employed in cohesive zone modeling (CZM), including bilinear, exponential, trapezoidal, and polynomial formulations, as illustrated in Figure 7.

By incorporating this behavior, CZM can simulate crack initiation and propagation without requiring an initial crack or separate crack-growth criterion. Carvalho and Campilho [36] verified various CZM law shapes for predicting the strength behavior of various adhesives in a single-lap joint configuration. Such validations are critical for ensuring the reliability of CZM parameters in design applications, as these parameters are highly dependent on the adhesive type and joint geometry [37,38]. Campilho et al. [39] developed a mixed-mode CZM tailored for ductile adhesives, applying it to stress and strength analysis of carbon-fiber reinforced polymer (CFRP) single-strap repairs. The model successfully captured the progressive damage and failure of the adhesive in repairs, aligning well with experimental results. In another work, Anyfantis and Tsouvalis [40] proposed a novel traction–separation law specifically for ductile adhesives that better captured mixed-mode behavior, leading to improved predictions for SLJs and double-strap joints under combined loading. 

One important aspect of CZM is determining the cohesive law parameters, including the initial stiffness, cohesive strength, fracture energy, and the shape of the softening curve. Campilho et al. [41] investigated the effect of cohesive law parameters in a triangular CZM, considering a thin adhesive layer in a single lap bonded joint. These parameters can be estimated using various techniques, such as the property identification method, direct method, and inverse method, each with its own level of complexity and accuracy. In subsequent work, Campilho and co-authors [42] examined different cohesive law shapes, including triangular, exponential, and trapezoidal, for thin adhesive layers. They found the triangular CZM to be most straightforward to use with fewer convergence issues in finite element simulations. Their study showed that the choice of CZM shape significantly influences results for joints using ductile adhesives and for joints with shorter overlap lengths, with the trapezoidal shape (which has a stress plateau after the peak before softening) providing the best correlation with experimental data. In contrast, for brittle adhesives, the CZM shape had minimal impact on the accuracy of strength predictions. Campilho and Fernandes [43] and Nunes et al. [44] noted that the triangular CZM underpredicted the strength of the highly ductile adhesive. This discrepancy is due to the limitations of the linear traction–separation law, which does not adequately capture the large deformation and energy dissipation capacity. They suggest that adopting a more representative cohesive law could improve predictive accuracy for such highly ductile adhesives.

**Figure 8:**
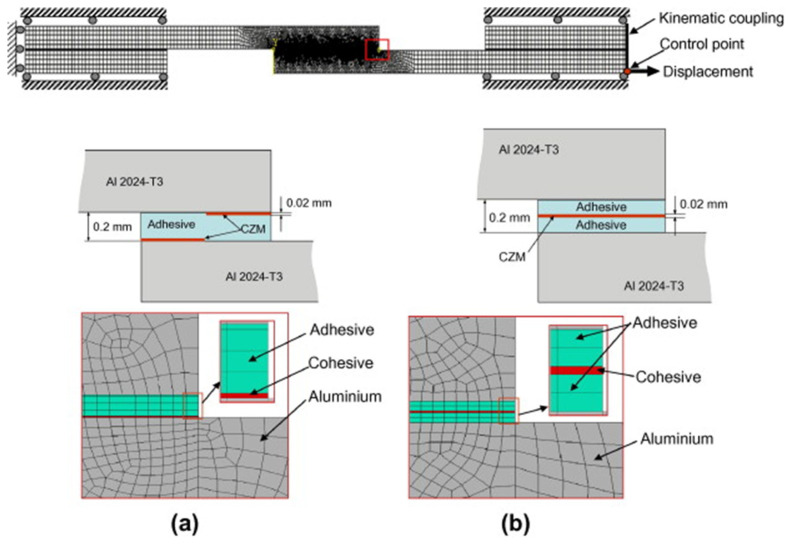
Positioning of the CZM element within the adhesive layer investigated by [45], (**a**) placing the CZM at the interface between the adhesive and the substrate, and (**b**) placing the CZM at the center of the adhesive layer.

**Figure 9:**
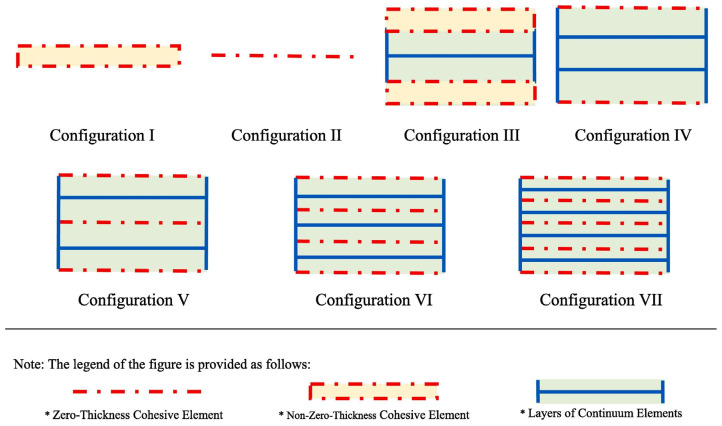
FE configurations for the adhesive layer modeling. Configurations I–VII represent different combinations of cohesive and continuum elements. Red dashed lines indicate zero-thickness cohesive elements; red blocks indicate non-zero-thickness cohesive elements, and blue blocks represent continuum elements [47].

A key advantage of CZM is that it does not require a priori knowledge of the crack path; the crack can initiate and propagate wherever the damage criteria are met. This is especially useful for complex joints where the failure path may not be obvious (e.g., in dissimilar adherend joints where one might develop asymmetric cracks). Sugiman et al. [45] studied the position of the CZM within the adhesive layer, as shown in Figure 8, and reported that whether centered or placed at the interface, it did not affect the predicted strength. However, based on their experimental observation of cohesive failure occurring within the adhesive, they chose to place the CZM within the adhesive layer rather than at the interfaces. Hartlen et al. [46] evaluated different cohesive element formulations within LS-DYNA for CZM of adhesively bonded interfaces, emphasizing that the choice of element type should align with the modeled adherend geometry and expected stress states. Mottaghian and Taheri [47] explored different finite element modeling strategies for the adhesive layers in a double-strap joint, as illustrated in Figure 9. It combines varying numbers of zero-thickness and non-zero-thickness cohesive elements with continuum elements. Configurations range from simple single-element models to more refined multi-element representations. They found that using multiple zero-thickness cohesive element layers provided the most accurate prediction of damage evolution in the joint. In fact, a configuration with five layers of these interface elements through the thickness gave the best results, capturing the progressive failure of the adhesive. Their study also considered geometric effects: increasing the strap length and thickness improved joint capacity, but only up to a point. Beyond a certain strap size, the joint strength could not be improved further, indicating that the load transfer had been maximized. 

Studies have consistently shown CZM to be effective for bonded joint simulations. For example, Campilho et al. [48] compared CZM and the XFEM in the analysis of single and double lap joints bonded with a brittle adhesive. It was mentioned that CZM demonstrated greater robustness and accuracy in capturing joint behavior. Sadeghi et al. [49] evaluated four FEM techniques (cohesive element method, surface-based cohesive method, XFEM, and VCCT) in 2D models of SLJs. They recommended using a trapezoidal traction–separation law instead of the simpler triangular model, as it offers more accurate predictions for the behavior of ductile adhesives. This is because a trapezoidal law can represent stable damage propagation rather than immediate softening after peak.

### 3.2. Extended Finite Element Method (XFEM)

XFEM enables crack propagation through finite elements by enriching their formulation to capture discontinuities, eliminating the need for predefined crack paths or cohesive interface elements. This flexibility is theoretically appealing for modeling adhesive joints, where the crack path is often unknown. However, its application to bonded joints has shown mixed success, as the crack growth may not accurately follow interface behavior or material boundaries. Sadeghi et al. [49] found that the XFEM technique gave inaccurate failure loads in thin-adhesive SLJs, while for higher thickness, it can be employed. De Sousa et al. [50] showed that XFEM was generally inadequate for capturing mixed-mode crack growth, whereas a standard CZM with a calibrated triangular law was highly accurate except in cases involving the highly ductile adhesive, which underpredicted the experimental results. In brittle joints, XFEM can be more acceptable, but even then, if multiple potential crack paths exist, XFEM requires additional criteria to decide the path.

In summary, XFEM can predict crack propagation in bonded joints without meshing the adhesive with cohesive elements, but it may need very fine meshes and is sensitive to how the crack initiation criteria are defined. It seems best suited for cases where the adhesive behaves in a predominantly brittle manner and for joints with thicker bondlines. For the thin bondlines, however, cohesive element approaches have been favored for reliability.

### 3.3. Virtual Crack Closure Technique (VCCT)

Virtual Crack Closure Technique (VCCT) is a fracture mechanics-based method used in finite element models to compute the energy release rate associated with crack growth. It operates by virtually closing a small crack extension and calculating the work required, assuming that this energy is equivalent to the energy needed for crack propagation. The method assumes linear elastic fracture mechanics (LEFM) and necessitates that the crack path follows element boundaries, requiring mesh alignment with the anticipated crack trajectory. Sadeghi et al. [49] included VCCT in their comparison and noted that while it can accurately predict crack propagation, it cannot capture crack initiation unless a pre-crack is assumed. The length of this pre-crack is typically defined based on the bond-line geometry, where damage is expected to be initiated. In practical terms, VCCT is highly effective for calculating energy release rates (G values) in scenarios with predefined cracks. However, it cannot predict initial failure loads in joints without an initial flaw, as it does not incorporate a damage initiation criterion.

### 3.4. Analytical Models for Failure Prediction

Analytical models have long been used to estimate failure loads by analyzing the elastic stress distribution and fracture behavior of the adhesive. Carrere et al. [51] proposed a two-stage failure criterion, first using an initial stress-based criterion for micro-crack initiation, followed by an energy-based criterion for crack propagation. Their model highlighted the need for large displacement considerations when predicting failure loads and emphasized the importance of capturing out-of-plane stress by using an adequate mesh, recommending at least 20 elements through the adhesive thickness for accuracy. Majidi et al. [52] developed the Point Stress (PS) criterion for double-strap joints. Instead of modeling cracks directly, they analyzed the elastic stress distribution along the adhesive mid-plane, taking into account the effective bond length of the joint. Similarly, Razavi et al. [53] introduced the Critical Normal Strain (CNS) criterion, which uses the strain distribution at the adhesive mid-line and defines failure when a critical normal strain over a certain distance is reached. It was demonstrated that the CNS parameters are independent of the selected reference joints and are also not influenced by the finite element mesh size. However, to determine the CNS constants, at least two joints with different bonding lengths are required. Both PS and CNS methods are limited to joints that remain mostly elastic up to failure, while they avoid the need for complex, full nonlinear simulations.

Some researchers have derived semi-analytical and closed-form solutions to predict adhesive joint behavior under complex loading. Yu et al. [54] developed a bond behavior model for CFRP–steel double-strap joints that accounted for both adhesive failure and steel yielding. Their model provided formulas to distinguish between elastic adhesive failure and plastic adherend failure cases. Additionally, the debond initiation location is also affected by whether the steel fails elastically or plastically. Although their formulations provide valuable insights, they assume a perfectly brittle adhesive. To address this limitation, they proposed a universal solution algorithm that could incorporate various adhesive constitutive models, making it adaptable to ductile adhesives as well. Li et al. [55] focused on predicting joint stiffness rather than strength. They developed an analytical method to predict joint stiffness based on shear stress–strain analysis in the bond region, independent of the bond length within practical design ranges.

### 3.5. 3D vs. 2D Plane Stress/Strain Models

When using 2D models for a joint of finite width, a choice must be made between plane stress and plane strain. Sadeghi et al. [49] compared plane stress and plane strain assumptions in their FE models and found that plane strain predicted a stiffer response and higher failure load than plane stress. This is because plane strain conditions constrain out-of-plane deformation, increasing the predicted joint strength. However, increasing the bondline thickness reduced this overprediction, allowing the plane strain models to provide a more realistic estimate of the failure load. In real adhesive joints, the stress state transitions from plane stress near the free edges to plane strain in the interior, driven by geometric and boundary constraints. The 3D models can capture this gradual transition and account for edge effects, often providing more conservative and realistic strength predictions compared to simplified 2D assumptions. Özer and Öz [56] and Rodríguez et al. [57] showed that a carefully built 2D plane-strain or plane-stress model can capture the critical stresses leading to failure. However, Heshmati et al. [58] found that 3D modeling provided more accurate predictions of ultimate joint strength compared to 2D modeling, primarily due to the non-uniform transverse stress distribution in the adhesive layer. In wide joints or situations with significant out-of-plane effects, 2D assumptions may misrepresent the stress state. Heshmati’s work on FRP–steel joints showed that 3D models captured the strength more accurately, and that imposing perfect symmetry in the model could prevent certain asymmetric failure modes from appearing, thus overestimating strength. In another approach [45,59], the adherends were modeled using 2D plane stress/strain elements, while the adhesive was modeled using 2D plane strain. It was shown that the mixed 2D scheme offered a closer match to actual 3D results. Therefore, while 2D CZM models are extremely useful and efficient for initial studies, one must be mindful of their limitations.

In conclusion, cohesive zone modeling (CZM) is the preferred method for simulating adhesive joint failure due to its ability to handle crack initiation and mixed-mode propagation. While XFEM and VCCT are useful in specific cases, they have not replaced CZM for most applications. Simplified criteria like PS and CNS offer engineers quick tools for design calculations, bridging the gap between basic calculations and full nonlinear simulations. However, validation against experimental data is essential, and models must account for real-world imperfections. Although 3D models offer more reliable results, well-calibrated 2D CZM models can achieve comparable accuracy in predicting joint strength, making them a cost-effective choice for design optimization when 3D simulations are computationally intensive.

Table 2 summarizes the modeling and simulation techniques discussed, considering their strength and limitations.

## 4. Material and Geometrical Modifications for Performance Enhancement

A great deal of research has focused on how modifying the joint’s materials or geometry can improve strength and durability. Ramezani et al. [60] reviewed recent advancements on the design and manufacturing of composite structure bonded joints, focusing on adherend modification techniques. These modifications aim to alleviate the high stress concentrations that often limit joint performance. Key strategies include tailoring adherend/adhesive geometry (e.g., tapers, recesses, fillets, notches), using hybrid or graded adhesives (bi-adhesive bondlines, functionally graded materials), and balancing adherend stiffness and embedded reinforcements. This section discusses several modifications and their effects.

### 4.1. Adherend and Adhesive Tapering with Fillets

**Figure 10:**
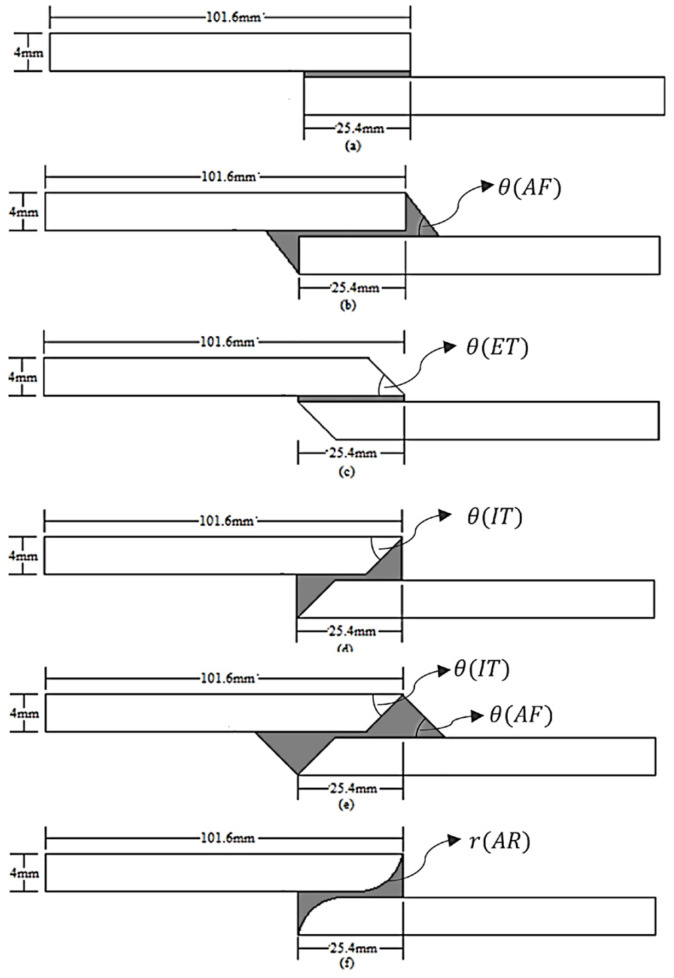
Geometry of single-lap joint (**a**) base configuration, (**b**) with adhesive fillet, (**c**) with external taper, (**d**) with internal taper, (**e**) internal taper with adhesive fillet, (**f**) with adherend rounding [62]. An external taper alone (**c**) had only a small effect, while the combination of internal tapering with adhesive fillet (**e**) was most effective, yielding the greatest reduction in stress concentrations and the largest improvement in failure load.

Tapering the adherend thickness at the overlap edges and adding adhesive fillets at the ends are well-established methods to reduce peel stress. By gradually diminishing the adherend thickness toward the edge (external or internal taper), the load path becomes less eccentric, and the stiffness transition is smoother. Fillets of adhesive at the ends create a curved transition for the adhesive layer rather than a sharp corner, which spreads out the peel stress. Taib et al. [61] demonstrated experimentally that the inclusion of spew fillets, along with optimized adhesive thickness and adherend stiffness, significantly reduced stress concentrations and improved joint strength in composite-based lap joints. Ejaz et al. [62] investigated several local geometric modifications in SLJs, as illustrated in Figure 10. Their results provide a clear illustration: among various SLJ modifications, an external taper alone had only a small effect (Figure 10c). In contrast, the combination of internal tapering with adhesive fillet was most effective (Figure 10e), yielding the greatest reduction in stress concentrations and the largest improvement in failure load. This suggests that modifying the adherend geometry at the overlap is more effective when coupled with adhesive fillets that smooth the adhesive geometry at the ends. However, special considerations are necessary when accounting for thermal effects. Da Silva and Adams [63] reported that in double-lap joints, the use of internal tapering combined with an adhesive fillet did not enhance joint strength when thermal stresses were significant. Moya-Sanz et al. [64] found that chamfering both the adherends and adhesive at 15° in composite SLJs was the most effective modification for boosting strength. The chamfered ends reduced eccentric load introduction and stress concentrations by providing a more gradual transition in cross-sectional geometry at the joint edges. The incorporation of spew fillets at the overlap edges is a well-established method for improving joint performance [65]. In contrast to joints with square ends, which exhibit pronounced stress concentrations at the overlap edges, spew fillets modify the joint geometry to distribute load transfer more gradually over a larger area. This results in a smoother load path and a more uniform shear stress distribution across the adhesive layer.

### 4.2. Notched Adherends

**Figure 11:**
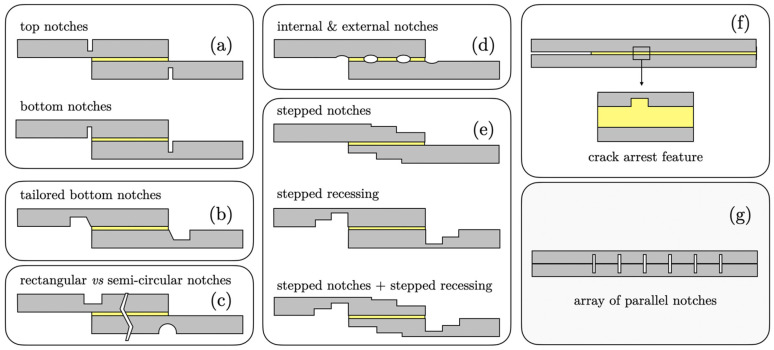
Schematic representation of various notched adherend geometries [68].

Introducing notches or cut-outs in adherends near the overlap ends can deliberately break a single long overlap into multiple shorter segments. This can relieve peak stresses in each segment by effectively restarting the load transfer in stages. Various notched adherend geometries are schematically represented in Figure 11. Kanani et al. [66] implemented notches in a dissimilar-material SLJ and combined that with a mixed-adhesive bondline. Their results showed that using a combination of a stiff and a ductile adhesive (mixed adhesive) led to a more uniform stress distribution and raised the failure load compared to a single adhesive. The presence of notches within the bonded area enhanced load transfer by breaking the overlap into smaller sections, which helped to relieve peak stresses in each segment. This effect was particularly beneficial in dissimilar joints, where the difference in adherend stiffness leads to asymmetric stress distribution. By increasing the number of notches, they achieved significant strength improvements for those hybrid joints. Moya-Sanz et al. [64] noted that for adherend recessing (thinning the adherend in the overlap region, creating a step), the depth of the recess was more critical than its length as it influences the reduction in cross-sectional area at the end and consequently lowers peak peel stresses, up to the point where the adherend might become too weak. Bahrami et al. [67] also found that notching the adherends adjacent to the overlap region can improve joint strength by reducing peel effects at the edges. Essentially, notches act as “crack stoppers” in design by breaking up what would be one large crack-driving region into smaller ones that are less prone to catastrophic propagation.

### 4.3. Adherend Thickness and Stiffness Matching

Ensuring both adherends in a joint have similar bending stiffness is beneficial. Hassan Vand et al. [69] used a numerical optimization (Bees Algorithm) to optimize the layup sequence of composite adherends in an SLJ, aiming to minimize adhesive stress. The optimal configuration was one where both adherends had high and nearly equal bending stiffness. When adherends were balanced in stiffness, the load was shared more evenly, and peak peel stress in the more flexible adherend was greatly reduced. Conversely, non-optimal configurations with a large stiffness mismatch led to the flexible adherend taking disproportionately more deformation and stress. In repairs or reinforcements, this might mean selecting a patch material similar in stiffness to the parent material or tapering a very thick patch down to reduce its stiffness at the ends. Abbasi et al. [70] studied composite SLJs and observed that increasing the adherend thickness raised shear stress in the adhesive; it actually reduced peel stress at the adherend surface. A thicker adherend can carry more load without bending, shifting the adhesive stress state closer to pure shear, which is favorable. However, beyond a certain adherend thickness, further increases yield diminishing returns, as the joint’s strength becomes limited by the adhesive’s shear capacity or by cohesive failure within the adhesive layer.

### 4.4. Bi-Adhesive Bondlines

**Figure 12:**
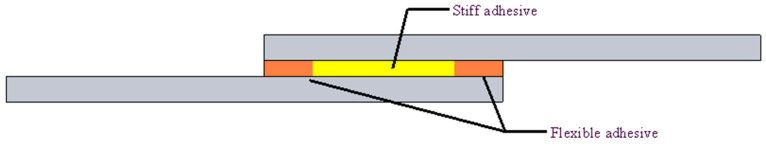
Schematic representation of a bi-adhesive SLJ.

In a bi-adhesive bonded joint, two adhesives with different properties are applied along a single overlap to optimize performance. Typically, a tough and compliant adhesive is used near the ends of the overlap, while a stiffer and higher-strength adhesive occupies the middle section, as shown in Figure 12. This configuration allows the stiff adhesive to carry the main shear load in the low-deformation region, whereas the flexible adhesive accommodates the larger peel and shear strains at the lap ends. This spatial tailoring helps achieve a more uniform stress distribution and a delay in failure onset. Özer and Öz [71] performed numerical and analytical analyses of bi-adhesive double lap joints and demonstrated that a well-chosen bond length ratio can, indeed, reduce peak stresses. Ramezani et al. [72] showed that a proper stiff–ductile adhesive pairing can significantly increase failure load by fostering a more uniform stress distribution along the overlap, validating the concept with DIC measurements. Fracture is generally observed to initiate at the transition zone between the stiff and compliant adhesives, with crack propagation occurring predominantly through the brittle adhesive layer. Nonetheless, after joint failure, the adherends remain partially bonded by the flexible adhesive. Jairaja and Naik [73] showed that in dissimilar adherend SLJs, a dual-adhesive bondline achieved higher strength than a single-adhesive bond due to improved stress distribution. Akhavan-Safar et al. [74] conducted a comprehensive review on the benefits and manufacturing challenges of using bi-adhesives. They emphasized that, while bi-adhesive joints can significantly enhance strength and reduce weight compared to single-adhesive joints, careful selection of a compatible ductile–stiff adhesive pair and appropriate joint geometry is crucial for optimal performance.

### 4.5. Functionally Graded (FG) Materials

Functionally graded adhesives and adherends have also been proposed to handle the peel stress problem. Instead of a sharp change from adherend to adhesive, a functionally graded material (FGM) can gradually transition either the adhesive’s modulus or the adherend’s properties. Stein et al. [75,76] studied analytical models for FG adhesive layers in SLJs with gradually varying adhesive properties through the bondline thickness, showing that grading the adhesive can smooth out stress peaks at the overlap edges and potentially delay failure. In a symmetric configuration, the adhesive modulus is distributed such that a soft adhesive at the ends deforms slightly to reduce peel stress, while a stiffer adhesive in the middle carries the shear load. Guin and Wang [77] studied FG adherends whose composition transitions from stiff at one end to flexible at the other. Their analytical model indicated that placing a stiffer material near the overlap and a more compliant material farther away reduced the stress concentration at the joint edges. In addition, combining FG adherends with adjustments to the adhesive, such as increasing adhesive thickness or decreasing its modulus, further helps in evenly distributing stress. FG materials eliminate abrupt interfaces between very different materials, thereby avoiding stress singularities. In practice, while manufacturing continuously graded adhesives or adherends is challenging, techniques like particle dispersion or 3D printing offer practical approximations.

### 4.6. Geometric Modifications and Load Distribution

Numerical studies have shown that geometric modifications such as changes in overlap length, adhesive thickness, or edge shaping can significantly influence stress concentration and load-bearing capacity [78]. Metehri et al. [79] numerically evaluated such effects in SLJS and demonstrated that optimized geometry can substantially improve tensile strength. Gultekin et al. [80] investigated the effects of adherend width and overlap length on the strength of adhesively bonded SLJs. They concluded that, for a constant bonding area, increasing the joint width resulted in a higher load-carrying capacity, a more significant effect than increasing the overlap length. Moreover, altering the bonding area from a rectangular to a square configuration further improved joint strength. In another study, Akpınar et al. [81] examined the influence of joint geometry on the strength of adhesively bonded joints with equal adhesive areas, focusing on step-lap, double-strap, and stepped double-strap configurations. Results showed that, for the same bonding area, incorporating both stepping and patching significantly increased displacement and load-carrying capacities. Together, these studies demonstrate that joint geometry, not only the total bond area, has a critical influence on joint performance and should be a key consideration in design.

In some applications, parts might not be flat due to design or repair considerations [82]. Instead, they could be pre-curved and then flattened onto a surface during bonding. Temiz et al. [83] and Akpinar [84] showed that using pre-curved adherends or patches can introduce compressive residual stresses at the overlap ends. These stresses, generated by spring-back after curing, effectively mitigated tensile peel stresses and increased joint load capacity. While effective, this strategy requires precise control, as excessive curvature could distort the joint or cause premature failure. Zhao et al. [85] conducted an experimental study on CFRP-to-steel double-strap joints with a modifiable peeling angle, allowing for controlled variation in the combination of shear and peel loading to investigate mixed-mode debonding behavior. Despite the geometric symmetry of the joint, the results showed that initial debonding occurred asymmetrically with interfacial failure developed between the CFRP strip and adhesive on one side, while CFRP delamination occurred on the opposite side. These findings suggest that designing joints to favor shear-dominant loading can significantly enhance the strength of adhesive-bonded joints.

### 4.7. Embedded Reinforcements and Surface Treatments

To improve stress distribution and damage tolerance in adhesively bonded composite joints, researchers have explored the integration of reinforcement layers, such as fabric meshes, veils, and textile plies within the adhesive layer or at the adhesive–adherend interface. These interlayers are particularly beneficial in composite adherends, where interface-driven failure dominates under tensile and fatigue loads. Taş and Soykok [86] studied S2-glass and Kevlar fabric interlayers in SLJs, demonstrating enhanced load capacity and more favorable failure modes. Morgado et al. [87] proposed embedding additional adhesive layers into CFRP adherends to mitigate delamination. Their experimental and numerical results showed higher strength and energy absorption, though delamination was not fully prevented. The adhesive’s strain-rate sensitivity played a key role in shifting failure modes. In another study, Carbas et al. [88] investigated fiber metal laminates (FMLs) by incorporating multiple aluminum sheets into CFRP composites to enhance through-thickness properties. The results showed that placing aluminum layers on the outer surfaces in an SLJ configuration effectively prevented delamination, resulting in the highest joint strength.

Nanoparticle-enhanced adhesives represent another approach to reinforcement. De Cicco et al. [89] reviewed the use of carbon, ceramic, and mineral nanoparticles to improve interlaminar shear strength and fracture toughness in FRP composites and bonded joints. Similarly, Jojibabu et al. [90] reported lap shear strength improvements of up to 50% with epoxy adhesives modified using carbon nanotubes, graphene nanoplatelets, nanoclay, nano-silica, and nano-alumina. While still under development, such interlayer and nano-reinforcement strategies show strong potential for enhancing the durability and reliability of bonded composite joints. Further optimization is needed regarding material compatibility, placement, and processing techniques. Paygozar and Sadigh [91] enhanced an epoxy-based adhesive by adding silica nanoparticles for use in aluminum double-strap joints. They observed the highest failure loads at an optimal nano-silica weight percentage, as the nanoparticles effectively hindered crack propagation. Ideally, both adhesive layers and straps should reach failure simultaneously, sharing the load until the final rupture. Saeimi Sadigh [92] demonstrated one way to achieve this simultaneous failure: by reinforcing the adhesive with nanoparticles. They added reduced graphene oxide (RGO) fillers to the main adhesive and found 30% higher ultimate load and greater joint deformation before rupture. Essentially, the reinforced adhesive became tougher, so it could stretch and carry a greater load than the neat adhesive. However, Marami et al. [93] reported that increasing the RGO content enhanced the ductility of the adhesive and resulted in a reduction in its ultimate tensile strength.

Adherend surface roughening typically increases surface area and mechanical interlocking, improving interface strength. However, extremely rough surfaces might trap air or create micro-notches. Usually, a moderate roughness combined with chemical treatments yields the best adhesion. Akpınar et al. [94] investigated the combined effects of adherend surface roughness and a nanostructure-reinforced adhesive, and observed a synergistic improvement in joint performance. The rough surface provided good wetting and interlocking, while the nanomodified adhesive had better mechanical properties. One caution is that too many nano fillers can increase the viscosity of the adhesive, which might harm the bond if dispersion is poor.

In an ideal joint, the adhesive would experience nearly uniform stress, leading to cohesive failure across a large section at once, rather than a crack initiating at one edge and propagating through. Techniques such as tapering, filleting, bi-adhesives, and prestressing aim to achieve this by promoting more even stress distribution. However, these methods must be applied with careful attention to trade-offs. Nevertheless, the literature demonstrates that these modifications can significantly enhance joint efficiency. Table 3 summarizes material and geometric modifications discussed, considering their benefits and limitations.

## 5. Defect and Failure Mode Analysis

Understanding how defects affect joint strength and how different failure modes initiate is vital for reliable design. Real bonded structures inevitably contain flaws such as adhesive voids, bondline porosity, unbonded areas, or damage in the adherends. Rather than assuming a perfect bond, researchers analyze defect sensitivity and failure progression to ensure joint designs are damage-tolerant.

### 5.1. Adhesive and Adherend Defects

#### 5.1.1. Adhesive Defects

**Figure 13:**
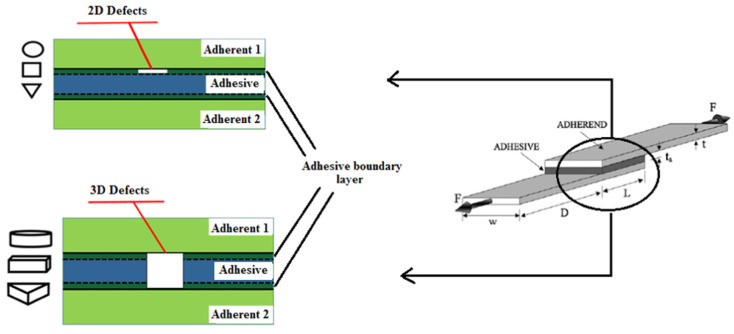
The 2D planar defects versus 3D volumetric voids in the adhesive joint [95].

**Figure 14:**
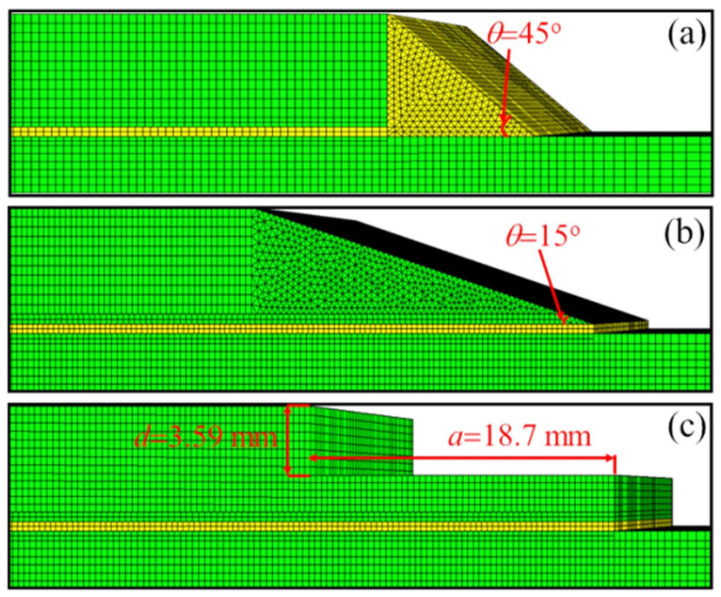
Various DSJ geometries investigated in [96], including (**a**) full adhesive chamfering, (**b**) adherend chamfering, and (**c**) adherend recessing.

**Figure 15:**
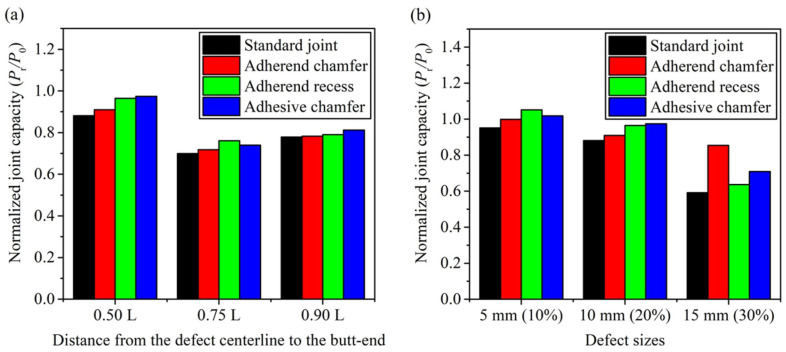
Effect of adherend chamfering, adherend recessing, and adhesive chamfering on damage tolerance of joints considering (**a**) defect locations and (**b**) defect sizes [96]. Normalized joint capacity is defined as the ratio of the failure load of the flawed joint to that of the defect-free control specimen.

Heidarpour et al. [95] conducted an experimental study on SLJs with intentional defects in the adhesive layer. They compared planar defects versus volumetric voids, as shown in Figure 13. The results clearly indicated that 3D voids led to a more pronounced reduction in joint strength compared to 2D planar defects. It was noted that strength dropped nearly linearly with increasing area of 3D void, but for 2D defects, the effect was nonlinear since small planar defects had little impact until they reached a critical size, after which strength fell sharply. Among different shapes of defects, circular voids were found to be the least detrimental for a given area. This suggests that if a defect is unavoidable, a smooth shape is less harmful than an irregular one. Fame et al.’s numerical investigations [96] on GFRP double-strap joints provided insight into the effects of defect size and location. They found that a single defect occupying less than 30% of the bonded area and located in the interior had minimal impact on peak stresses. The joint could essentially redistribute the load around the internal flaw. However, defects located near the overlap ends or the presence of more than two small defects in combination significantly increased local stress concentrations and reduced joint strength. This suggests that some level of defect is tolerable in the interior of a joint, but defects at critical high-stress regions or too many defects can sharply reduce strength. They also compared the damage tolerance of various defective joint geometries, including adherend chamfering, adhesive chamfering, and adherend recessing, as shown in Figure 14. The results, illustrated in Figure 15, demonstrate that adhesive chamfering offers the greatest improvement in damage tolerance, followed by adherend recessing and then adherend chamfering. However, when defects exceed 30% of the bonded area, adherend chamfering provides the highest damage tolerance. Wu et al. [97] proposed a design framework to account for defects in bonded joints by considering the detectability of defects via non-destructive testing (NDT). In their concept, if a defect is large enough to be found by NDT, the design should consider it in the strength calculations. If it is below the detection threshold, the joint is treated as structurally intact. Their framework was demonstrated on joints with circular defects and relatively short bond lengths. Therefore, further work is needed to generalize it to arbitrary defect shapes and long overlaps.

#### 5.1.2. Adherend Defects

Composite adherends can have hidden defects such as delamination. Panigrahi [98] investigated SLJs with composite adherends containing pre-existing delamination positioned at similar locations near the joint region. Interestingly, the delamination in the bottom adherend grew more than the top adherend’s delamination. Using fracture mechanics, they found that Mode I (opening) forces were the primary driver for delamination propagation, more so than Mode II (shear) or Mode III (tearing).

### 5.2. Failure Mode Transitions

The failure mode of bonded joints is highly influenced by adhesive type and joint configuration, and can be strategically altered to improve performance through careful material and geometric selection. Mohabeddine et al. [99] bonded CFRP–steel double strap joints with either a rigid brittle or a ductile tough adhesive; with the tough adhesive, the joints achieved higher ultimate loads and failed only by CFRP delamination, whereas the brittle adhesive joints failed with a combination of cohesive failure and delamination. Tough adhesives thus improve damage tolerance and are more suitable for such joints. While joints bonded with ductile adhesives can reach considerably higher strength than using brittle ones [100], for highly ductile adhesives, increasing the adhesive layer thickness can reduce SLJ strength [99]. A thicker ductile layer allows for more rotation and peel deformation, offsetting the benefit of its toughness. This underscores that each parameter (adhesive thickness, overlap length, adherend stiffness, etc.) has an optimum value rather than “more is always better”. Barbosa et al. [101] emphasized that the optimal joint type can depend on the adhesive’s properties. For joints that inherently have nonuniform stress, a ductile, lower-strength adhesive is beneficial to relieve peaks by local yielding. In such cases, configurations like scarf or double-lap joints are ideal, as they enable full utilization of the adhesive’s strength while minimizing stress concentrations. Thus, the failure mode can be “tuned” by matching adhesive to joint type. If adhesive failure is observed, switching to a tougher adhesive may shift failure into the adherend. Conversely, if premature adherend failure occurs, the adhesive may be overly strong, and a more compliant adhesive could promote better load distribution. This underscores that joint configuration and adhesive type must be considered together.

The observation by Azeem et al. [33] that mismatched double-strap joints had one side fail slightly before the other, providing higher overall toughness, suggests a principle of **Designing for controlled failure**. In some structures, you might intentionally allow one element to yield first so the structure can redistribute the load. In bonded joints, it is tricky because, usually, one wants both sides to carry equal load. But if a slight asymmetry can be introduced without compromising peak load, it might make the joint more tolerant to a defect on one side.

### 5.3. Surface Texture and Its Impact on Failure Modes

**Figure 16:**
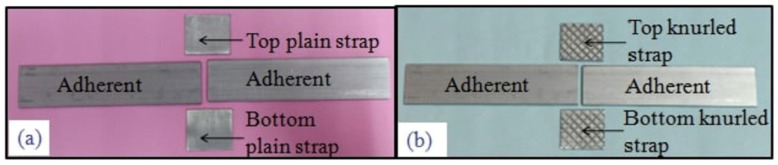
Types of straps used in a DSJ configuration for this study [102]: (**a**) plain strap, (**b**) knurled strap.

**Figure 17:**
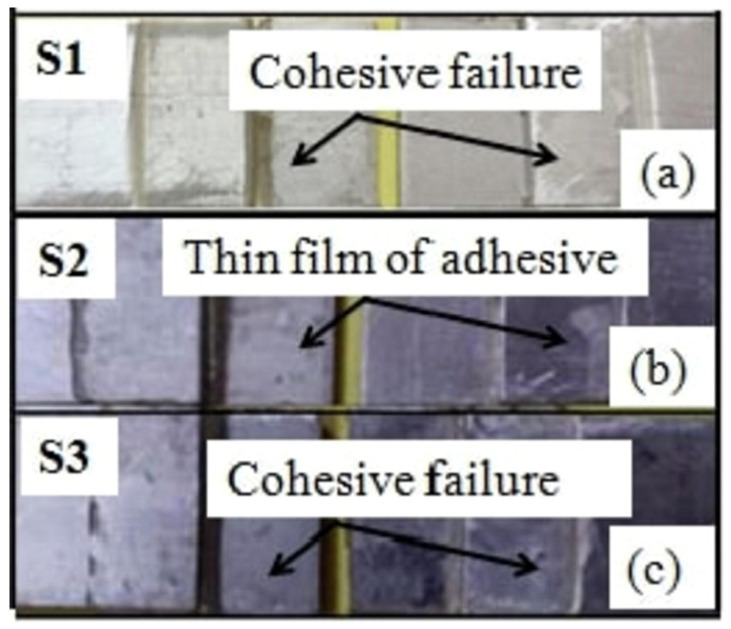
Experimentally obtained failure modes considering plain straps [102]: (**a**) cohesive failure in the adhesive, (**b**) adhesive failure due to poor surface preparation, (**c**) cohesive failure in the adhesive.

**Figure 18:**
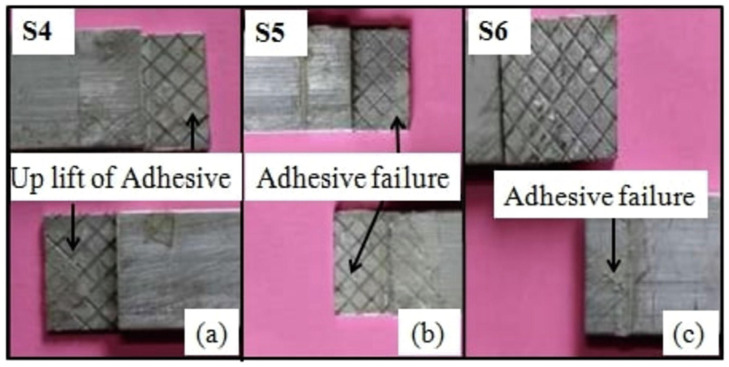
Experimentally obtained failure modes considering knurled straps [102]: (**a**) uplift of adhesive due to improper adhesive preparation on the adherend, (**b**) adhesive failure, (**c**) adhesive failure.

Sahana’s experiment [102] is an example of how a modification flips the mode. They considered both plain and knurled straps in a DSJ configuration, as shown in Figure 16. Joints with plain straps primarily failed cohesively within the adhesive, whereas those with knurled straps failed at the adhesive–strap interface (adhesive failure) as shown in Figure 17 and Figure 18. The knurled texture, intended to improve mechanical interlocking, led to premature interfacial failure, which is generally undesirable as it indicates that the adhesive did not reach its full strength. Thus, while surface roughness can sometimes improve bond strength, excessive roughness might introduce stress concentrations or air pockets that weaken the interface.

### 5.4. Fatigue Behavior Under Repeated Tensile Loading

In practical applications, adhesively bonded joints are often subjected to cyclic tensile loading, leading to progressive damage accumulation, interfacial debonding, or cohesive failure over time. Malekinejad et al. [103] have reviewed strategies to enhance the fatigue strength of adhesively bonded composite joints. Fatigue performance is influenced by several factors, including adherend thickness, adhesive stiffness, joint geometry, and defect sensitivity. Calabrese and Vassilopoulos [104] reported significant differences in fatigue resistance between thin and thick bonded composite joints, emphasizing the importance of structural compliance. Similarly, Wong et al. [105] studied composite–metal joints and observed slow-growth delamination and disbond propagation under fatigue loading, highlighting the need for early damage detection strategies. Sekiguchi and Sato [106] examined how bondline thickness influences fatigue resistance and found that increasing the adhesive thickness enhances fatigue performance by improving fracture toughness and reducing crack growth rates, particularly under low-cycle loading conditions.

Hybrid joining approaches also offer potential benefits in fatigue-critical applications. Gamdani et al. [107] demonstrated that combining multiple bolts with adhesive bonding in composite laminates improved load-sharing and mitigated premature failure under tensile loading, findings that are particularly relevant to fatigue-prone structures.

While fatigue is not always the primary design constraint in static-dominant structures, it becomes critical in aerospace, wind turbine, or automotive applications where vibration and load cycling are prevalent. The incorporation of graded adhesives, spew fillets, or toughened interlayers has shown potential to delay fatigue crack initiation [108,109]. However, more integrated modeling approaches are needed to capture fatigue degradation, especially for mixed-mode and thick adhesive joints.

## 6. Comparative Performance of Different Joint Types

**Figure 19:**
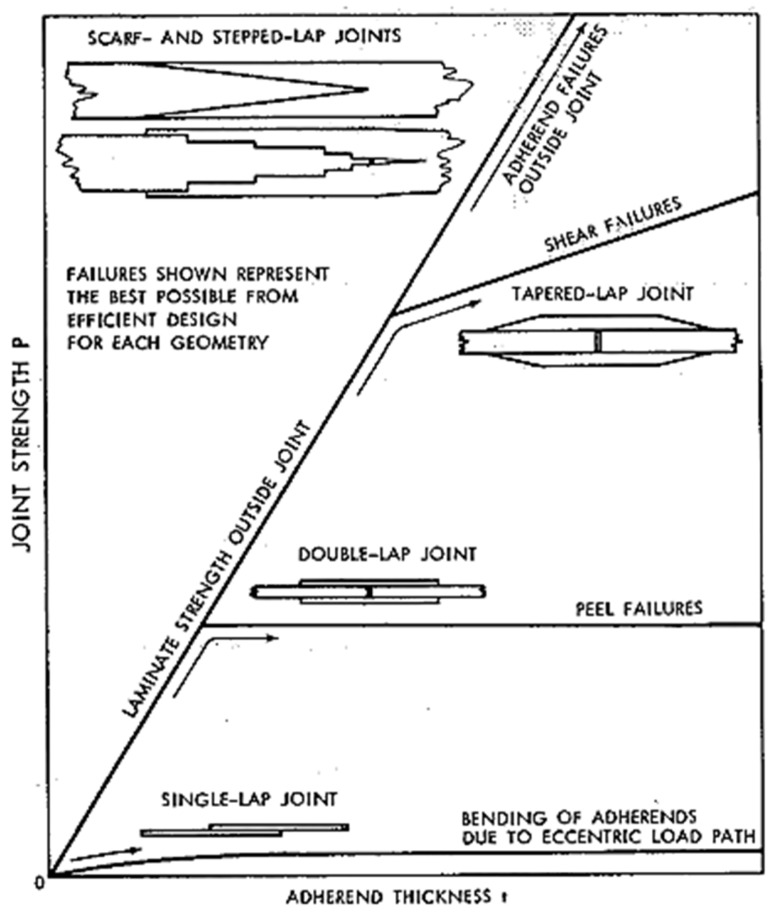
Influence of joint size on selection of joint configuration [110]. SLJs showed the lowest strength, while scarf and stepped joints demonstrated highest strength.

Given the variety of joint configurations and enhancements available, a natural question is, Which joint design performs best under a given scenario? The answer often depends on multiple factors, including the adherend and adhesive properties and the loading conditions. Hart-Smith [110] presented a comparative analysis illustrating how adherend thickness influences the selection of optimal joint configurations to achieve a desired joint strength. This study encompassed single/double, tapered, stepped, and scarf joints, as depicted in Figure 19. For thin adherends, most configurations yielded comparable joint strength, apart from SLJs, which consistently showed the lowest strength. As adherend thickness increased, alternative configurations demonstrated superior performance. It is important to note that Hart-Smith’s analysis focused on general design principles and did not account for the specific effects of material properties or environmental and operational conditions. Nonetheless, that study offers valuable general insights into joint configuration selection.

Building on these general principles, the section examines specific joint configuration comparisons in more detail:

### 6.1. Double-Strap vs. Single-Lap Joints

Double-strap joints are typically stronger and more damage-tolerant than single-lap joints. The symmetric load introduction of double straps avoids the severe bending of single laps, leading to higher load capacity for a given adhesive. For example, Fame et al. [96] found that double-strap joints generally exhibited superior damage containment, as a crack in one adhesive layer did not immediately fail the joint because the other layer still carried load.

### 6.2. Stepped-Lap vs. Single-Lap Joints

Stepped-lap joints (multiple smaller overlaps in series) generally outperform single laps because they increase the bonded area without a large eccentric offset. Silva et al. [111] compared stepped-lap to single-lap joints and found that stepped joints had higher strength, especially at longer overlap lengths. The stress distribution in a stepped-lap is more favorable since, essentially, a series of mini-laps means the load is introduced gradually through each step, avoiding a single high-stress location. However, given the simpler manufacturing process of single-lap joints, the final selection should balance performance benefits with fabrication complexity.

### 6.3. Double-Butt Lap vs. Single-Lap Joints

Goudarzi and Khedmati [112] reported a case where a single-lap joint outperformed a double butt-lap joint using the same adherend materials. The single-lap’s flexibility allowed for more energy absorption, whereas the double butt-lap, being stiffer, failed at a lower overall load but in a more brittle fashion. This highlights that a stiffer joint is not always better if it leads to low ductility; sometimes, adding supplemental flexibility can increase ultimate load by allowing for redistribution.

### 6.4. Weight Efficiency

When comparing different joint types, it is not only about absolute strength; it is also about efficiency. A scarf joint may require a long overlap due to its shallow taper, which can increase material use and joint length, though it can provide high strength. A stepped-lap joint increases weight by overlapping material in multiple steps, adding thickness and potentially complicating manufacturing. If a single-lap can achieve the required strength with a tough adhesive, it might be lighter and simpler than a stepped-lap joint [111]. Therefore, it is recommended to use a joint efficiency metric that considers both adherend strength and weight as a design criterion, rather than relying solely on absolute strength.

What the literature provides is guidance on how various bonded joint types behave under tensile loading, along with design adjustments that can enhance their performance:

- If using a brittle high-strength adhesive and aiming to maximize joint strength, a double-lap or scarf joint is preferable, as these configurations allow for more effective utilization of the adhesive’s load-carrying capacity. But one must ensure high manufacturing precision for alignment and bondline thickness;
- If a ductile adhesive is used or ease of fabrication is desired, a single-lap joint with appropriate adherend modifications can be an unexpectedly effective solution. Single-lap joints remain attractive for many structures due to their simplicity and the fact that many modifications (taper, fillet, etc.) can mitigate their weaknesses;
- Stepped-lap joints offer a middle ground: their multiple overlapping steps generally achieve higher strength than single-lap joints by distributing the load over a larger bonded area and reducing peak stresses. However, this performance gain comes at the cost of increased fabrication complexity and added weight;
- Double-strap joints are an excellent choice for repairing or joining structural members when both sides are accessible. They are less sensitive to adherend thickness mismatch than single-lap joints, since each adherend is bonded on both sides, minimizing bending effects. These joints significantly reduce peel stress and double the bonded area for essentially the same overlap length, though this comes with the trade-off of increased weight.

**Neither adhesive nor geometry alone is the answer; it is the combination that matters**. Modern design approaches often employ optimization algorithms that can pick the best combination of overlap length, adherend geometry, adhesive type, etc., for a specific application. 

In conclusion, from a comparative perspective, designers should aim to use the simplest joint configuration that meets performance requirements, enhancing it with material and geometric modifications when necessary. Hybrid joint strategies, which combine adhesive bonding with mechanical fasteners, also represent a promising avenue for improving structural integrity and damage tolerance [113,114]. These configurations can offer increased load capacity and more progressive failure modes by leveraging the complementary strengths of adhesive shear transfer and bolt clamping forces. However, the effectiveness of hybrid joints depends on a careful balance since if the mechanical fastener is significantly weaker than the bonded connection, an overly strong adhesive may not enhance the overall joint performance [115].

## 7. Discussion

This review has synthesized a wide range of research concerning the tensile behavior of adhesively bonded joints. The performance of these joints is dictated by a complex interplay of geometric configuration, material properties, stress distribution, defect sensitivity, and failure mechanisms. A comparative analysis of commonly used configurations, including single-lap joints (SLJ), single-strap joints, and double-strap joints, reveals key performance trade-offs. SLJs, while simple and widely used, are prone to peel-induced failure due to their inherent eccentric loading path. Conversely, double-strap joints benefit from structural symmetry, reducing peel stress and improving load distribution, although they require more adhesive and structural weight.

Several geometric modifications have demonstrated effectiveness in enhancing joint strength by reducing stress concentrations. Strategies such as adherend tapering, fillet formation, stepped bonding, and the use of notched geometries redistribute load more uniformly and suppress stress peaks at the overlap edges. Particularly for asymmetric joints like SLJs, these modifications often shift the failure mode from brittle interfacial failure to more favorable cohesive or distributed damage modes.

On the material side, tailoring adhesive properties has a profound influence on joint behavior. The integration of bi-adhesive bondlines (using compliant adhesives near the ends and stiff adhesives in the middle) has been shown to improve damage tolerance and delay crack initiation. Functionally graded adhesives and nano-enhanced adhesives represent emerging solutions to reduce interfacial mismatch and boost fracture toughness. In parallel, the use of hybrid adherends or patch materials with matched stiffness further enhances performance by balancing deformation across the joint.

Numerical modeling has been widely used for adhesive joint design. Cohesive zone modeling (CZM) stands out as the most widely adopted simulation tool due to its capability to capture mixed-mode failure without requiring predefined crack paths. Accurate modeling, however, depends on reliable calibration of cohesive law parameters such as fracture energy and peak traction. Advanced techniques, including inverse methods and digital image correlation (DIC)-assisted validation, are increasingly used to refine model inputs. While extended finite element method (XFEM) and virtual crack closure technique (VCCT) approaches offer benefits in specific fracture mechanics contexts, their application remains more limited compared to CZM, particularly in joints involving complex adhesive behavior or non-linear deformation.

This review also identifies how joint performance degrades in the presence of defects. Void shape, location, and size are critical, with edge-located flaws having a more detrimental effect than internal ones. Moreover, defect-tolerant design strategies such as adhesive chamfering or distributed reinforcement can mitigate performance loss. The interplay between defect geometry and joint modifications highlights the importance of integrated design.

Finally, failure mode transitions can be intentionally controlled through appropriate material and geometric selections. For example, switching from brittle to ductile adhesives or adjusting bondline thickness can shift the failure mode from interfacial debonding to stable cohesive failure. This tunability allows engineers to design joints that are not only strong but also fail safely and predictably.

## 8. Conclusions and Future Prospects

This review has presented a comprehensive synthesis of recent advances in the design, modeling, and optimization of adhesively bonded joints under tensile loading. Drawing from experimental findings, simulation studies, and analytical models, several important insights have emerged regarding how to improve joint performance and predict failure mechanisms effectively:

- Joint performance is governed by the interplay of geometry, material properties, and loading conditions. Among the configurations reviewed, double-strap joints generally exhibit higher strength and damage tolerance due to their symmetric structure, while single-lap joints, though simple, are more prone to peel-induced failure because of their inherent eccentric loading;
- Stress concentration remains the primary cause of failure in adhesive joints. Strategies such as adherend tapering, inclusion of fillets, and the introduction of notched geometries have been consistently shown to redistribute stresses more evenly across the bondline and reduce peak values at critical regions;
- Modeling techniques, particularly CZM, have proven highly effective in capturing progressive failure behavior, especially in mixed-mode loading scenarios. CZM enables crack initiation and propagation prediction without predefining crack paths, making it more adaptable to complex geometries and material systems;
- The failure mode of bonded joints can be controlled through the careful selection of adhesive type and joint design. For example, replacing a brittle adhesive with a ductile one or optimizing bondline thickness can shift the failure from an interfacial debonding to a stable cohesive mode, improving overall reliability.

These findings provide a solid scientific foundation for guiding the design and analysis of adhesively bonded joints. Building upon these scientific insights, several practical strategies can be proposed to guide applied engineering designs:

- Tailoring adhesive properties, for instance, using bi-adhesive or functionally graded bondlines can significantly improve damage tolerance, reduce stress concentrations, and delay crack initiation, especially near overlap edges;
- Balancing adherend stiffness is essential for minimizing differential deformation and ensuring more uniform stress distributions across the adhesive. This is particularly critical in joints involving dissimilar materials or patch reinforcements;
- Material enhancements, such as nano-reinforced adhesives or embedded interlayers, have demonstrated potential in improving load-carrying capacity, energy absorption, and resistance to fatigue and environmental degradation;
- Geometric modifications, including tapering, fillets, stepped bonding, and pre-curved adherends, help suppress localized failure and can significantly enhance joint strength without dramatically increasing manufacturing complexity;
- Simulation and modeling tools, especially when validated with experimental data, should be incorporated early in the design process to predict performance under different load cases and to identify optimal configurations for weight, strength, and durability.

**Figure 20:**
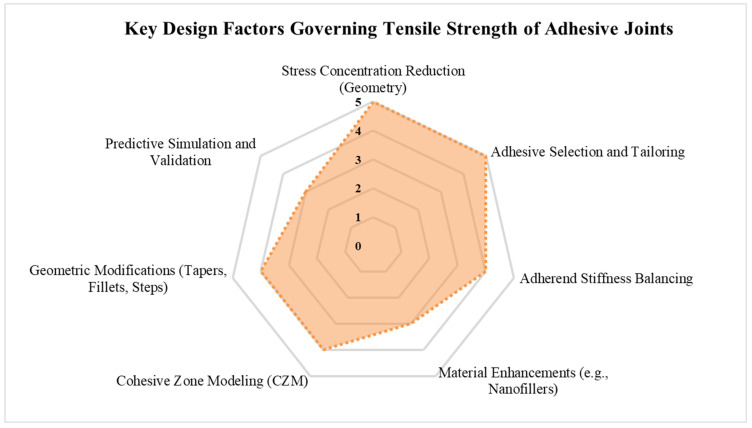
Radar chart illustrating the relative importance of key design factors influencing the performance of adhesively bonded joints under tensile loading. Scores are derived from a synthesis of literature findings, where a value of 5 represents the highest impact on joint strength, stress distribution, or failure mode control.

To provide a visual summary of the reviewed findings, a radar chart is presented in Figure 20, highlighting the relative importance of key design factors influencing the performance of adhesively bonded joints. 

Future research will likely concentrate on hybrid approaches, integrating real-time structural health monitoring with adaptive design features such as self-healing adhesives or damage-detecting materials and expanding these advancements to address additional loading scenarios. By ensuring the long-term reliability of joints throughout their service life, engineers can fully leverage the advantages of adhesive bonding, such as lightweight design and distributed load transfer, while effectively addressing its associated challenges. The reviewed literature offers a solid foundation for future advancements in bonded joint design and technology.

## Figures and Tables

**Table 1:** Summary of key characteristics and design considerations for joint configurations studied.

Joint Type	Key Characteristics	Design Influences	Failure Modes	Performance Optimization
Single-lap joint (SLJ)	Eccentric load path induces peel and shear stress; sensitive to secondary bending	Overlap length [10,11], adherend stiffness/thickness [12,13], adhesive layer thickness [19], material mismatch [14,15]	Cohesive, interfacial, adherend delamination/yielding, mixed-mode depending on adherend/adhesive properties [9,16,17,18,19]	Use of stiff, similar adherends [14,15]; minimize peel stress via geometry or stacking sequence adjustments [16,17]
Single-strap joint	Two adherends with strap on one side; asymmetric about adhesive plane, induces bending moment	Strap/adherend geometry (tapered ends) [20,21], adhesive thickness [22], fiber orientation [22], overlap length [20,22]	Adhesive failure, interfacial debonding, edge stresses [21,22]	Tapered adherend/strap ends [21], optimal bondline thickness [22], fiber alignment with load direction [22]
Double-strap joint	Straps on both sides, symmetric configuration, eliminates eccentricity and peel stress	Effective bond length [24,25], strap/adherend thickness [26,27,28], layup orientation [30,31,32], adhesive/adherend material properties [28,31,32]	Cohesive failure, interfacial debonding, delamination depending on layup and material choice [29,30,31,32,33]	tailored layups for load path [30,31,32], avoid intermediate inserts [27], model dynamic behavior accurately [34]

**Table 2:** Modeling and simulation techniques studied for adhesively bonded joints.

Modeling Technique	Key Features	Strengths	Limitations	Case Strategies
Cohesive Zone Modeling (CZM)	Uses traction–separation law to simulate progressive damage and failure; does not require pre-crack	Captures mixed-mode behavior, works well with ductile/brittle adhesives; validated by experiments [36,39,40,41,42,43,44,48,49]	Requires accurate parameter calibration; shape of CZM law affects accuracy, especially for ductile adhesives [36,39,40,41,42,43,44]	requires element choice and cohesive zone placement tailored to geometry and failure mode [45,46,47]
Extended Finite Element Method (XFEM)	Allows cracks to propagate without mesh alignment or predefined paths	No need for predefined crack paths; useful in brittle joints [49,50]	Less accurate in mixed-mode or ductile failure; sensitive to mesh and crack initiation criteria [49,50]	Brittle adhesive joints with thicker bondlines; less reliable for thin adhesive or ductile failure
Virtual Crack Closure Technique (VCCT)	Computes energy release rate using LEFM principles; requires predefined cracks	Accurate prediction of crack propagation path when initial crack is known [49]	Cannot simulate crack initiation; requires mesh-aligned crack path and LEFM assumptions [49]	Fracture propagation with predefined cracks, most effective for brittle interfaces or layered composite materials
Analytical Models	Predicts failure based on stress or strain distribution without full FE simulation	Quick failure estimates; independent of mesh size; useful in early design stages [52,53]	Limited to elastic range; not suitable for highly nonlinear or ductile adhesive behavior [51,52,53,54]	Preliminary joint design; simple bonded joints; educational/parametric studies
2D plane stress/strain vs. 3D Modeling Approaches	2D uses plane stress/strain; 3D includes edge and transverse effects	2D efficient and accurate for narrow joints; 3D captures full geometry [45,49,56,57,58,59]	2D may misrepresent stress results; 3D is computationally expensive [49,58]	2D for initial optimization; 3D for final verification; Mixed 2D plane stress/strain modeling

**Table 3:** Summary of material and geometrical modifications studied for performance enhancement.

Modification Type	Key Features	Benefits	Limitations
Adherend and Adhesive Tapering with Fillets	Tapered adherends and adhesive fillets reduce stress concentrations by smoothing transitions	Lower peel stress; improved load transfer; increased joint strength [61,62,64]	Less effective under thermal stress [63]; requires precise geometry and process control
Notched Adherends	Deliberate notching breaks long overlaps into multiple segments to spread stress	Reduced peak stress [67]; increased strength in dissimilar joints [66]	May weaken adherend locally; needs precise notch placement [67]
Adherend Thickness and Stiffness Matching	Balances load between adherends; reduces stress on more flexible side	Improved load sharing; reduced peel and bending [69,70]	Limited by adhesive strength; diminishing returns beyond optimal thickness [70]
Bi-Adhesive Bondlines	Combines stiff and ductile adhesives along overlap to tailor stress distribution.	Delays failure; raises joint strength; improves load distribution; weight-sensitive applications [71,72,73,74]	Crack may initiate at adhesive interface [72]; manufacturing complexity [74]
Functionally Graded Materials (FGMs)	Gradual transition of modulus in adhesive or adherend to reduce stress gradients	Smooth stress variation; avoids singularities; higher strength [75,76,77]	Manufacturing challenges; limited real-world use [77]
Geometric Modifications and Load Distribution	Overlap length, width, curvature, and patch shape affect load transfer efficiency	Enhanced load capacity; reduced edge stress; tailored load paths [79,80,81,83,84,85]	Requires accurate modeling and prototyping [83,84]
Embedded Reinforcements and Surface Treatments	Interlayers, nanoparticles, or surface roughening enhance stress transfer and toughness	Higher joint strength; improved toughness and durability [86,87,88,89,90,91,92,93,94]	Potential dispersion/viscosity issues; requires control of filler amount [89,94]

## Data Availability

No new data were created or analyzed in this study. Data sharing is not applicable to this article.

## Abbreviations

The following abbreviations are used in this manuscript:

3D	Three-dimensional
CFRP	Carbon-fiber-reinforced polymer
CNS	Critical Normal Strain
CZM	Cohesive zone modeling
DIC	Digital Image Correlation
DSJ	Double-strap joint
FGM	Functionally graded material
FML	Fiber–metal laminate
GFRP	Glass-fiber-reinforced polymer
LEFM	Linear elastic fracture mechanics
NDT	Non-destructive testing
PS	Point Stress
RGO	Reduced graphene oxide
SLJ	Single-lap joint
VCCT	Virtual crack closure technique
XFEM	Extended finite element method
